# Biodegradable Polymer Films with a Natural Antibacterial Extract as Novel Periodontal Barrier Membranes

**DOI:** 10.1155/2019/7932470

**Published:** 2019-08-14

**Authors:** Zehra Betul Ahi, Nergis Zeynep Renkler, Mine Gul Seker, Kadriye Tuzlakoglu

**Affiliations:** ^1^Yalova University, Department of Polymer Engineering, Yalova 77200, Turkey; ^2^Gebze Technical University, Department of Molecular Biology and Genetics, Kocaeli, Turkey

## Abstract

Biodegradable composite membranes containing propolis were produced from PCL/PLLA blends using a simple and low-cost solvent casting method, and subsequently their physicochemical, mechanical, and antibacterial properties were characterized. SEM analysis revealed that the addition of propolis has created honeycomb-like structures on the film surfaces. The flexibility of the films increased in the presence of propolis, which may provide ease of use during application. Propolis disrupted the organized structure of both polymers at the molecular level and caused decreases in the melting points. The films with propolis showed faster degradation in physiological conditions due to this molecular disruption. Moreover, the PLLA/PCL/propolis composite films exhibited remarkable antibacterial activities against *S. aureus*. Collectively, the data suggest that the produced films might be used as an alternative to exiting barrier membranes in guided tissue regeneration.

## 1. Introduction

Periodontitis is a periodontal inflammatory disease that irreversibly destructs the tooth supporting tissue. The loss in dental tissue progresses the loss of attachments, periodontal pocket formation, increased mobility, and loss of the alveolar bone. In severe cases, it can result in tooth loss [1]. The basic aim of the treatments is the reconstruction of damaged supportive tissue, periodontal ligaments, bone tissue, or, in the other words, the entire defect [2, 3]. The effectiveness of a variety of techniques, including root surface conditioning, coronally positioned flaps, and bone replacement grafts, has been tested and evaluated [4–6]. However, these procedures have limitations in attaining complete and predicable regeneration, especially in advanced periodontal defects. To overcome the limitations, guided tissue regeneration (GTR) therapy has been introduced to the clinic as it is a well-established procedure and shows successful outcomes to regenerate damaged periodontal tissue [7, 8]. The main purpose of GTR is to establish a boundary between epithelial cells that produce periodontal ligament and alveolar bone cells with assistance of a barrier membrane made from various materials. This membrane is utilized to promote the increase in selected cell populations that facilitates the periodontal regeneration and to prevent the migration of undesirable cells into the wound area. Thus, the epithelial tissue cells are not allowed to migrate through the defect site and to provide sufficient space for new bone formation.

The commercially available GTR membranes are fabricated from biodegradable and nonbiodegradable polymers. Most of nonbiodegradable membranes in the market are made of polytetrafluoroethylene (PTFE), a synthetic polymer, whereas collagen is the most commonly used biodegradable and natural material in clinically available biodegradable membrane production [9]. Nevertheless, several drawbacks have been reported for both types [9, 10]. Commercial nonbiodegradable PTFE membranes require a secondary surgery for their removal which may injure and compromise the obtained regenerated tissue [11]. On the contrary, collagen-based membranes permit a single-step procedure, but they may fail before complete healing due to their low mechanical properties compared to the synthetic polymers. Besides that, the loss of space-maintaining ability in humid conditions, risks of a disease transmission, and too rapid biodegradation are the other drawbacks of these membranes [12]. As an alternative to the collagen membranes, synthetic biodegradable polymers, such as PLLA, PLGA, PCL, and their copolymers, have been used to make commercial biodegradable GTR membranes [13]. Although they are not as biologically active as collagen, their controllable biodegradability, processability, and good handling properties render them to widely consider as a membrane material both for *in vitro* experiments and the clinic.

Biocompatibility, cell selectivity, space formation, tissue integration, ease of use, and biological activity are the basic criteria required in the design of GTR barrier membranes. Ease of application, maintaining the stability desired period of time during recovery, easy sterilization, and preventing bacterial formation can be also added to this list as superiorities in the selection of a GTR membrane. Particularly, antimicrobial activity becomes more essential in order to inhibit possible microbial colonization caused by the exposure of the membrane.

Clinically, local or systemic administered antibiotics are still the first choice to avoid the postoperative infection after periodontal surgery. However, they have clinical limitations due to the emergence and increase of antibiotic-resistant bacteria and carry the risks of drug-induced hepatotoxicity. The use of biomaterials with antimicrobial properties gains an increasing interest in periodontal treatments. Propolis, which is a natural material made by honey bees and composed of plant nectars, has numerous useful features including antimicrobial, anti-inflammatory, antioxidant, antimanic, and carcinostatic effects as well as the ability to induce regeneration in various tissues such as the bone, cartilage, and dental pulp [14–16] The raw propolis has more than 300 identified constituents, which are classified as resins (50%), waxes (30%), essential oils (10%), pollen (5%), and various organic compounds (5%) [17–19]. Its strong antibacterial effect is attributed to the phenolic compounds of the flavonoid fraction within the structure [20, 21].

Therefore, the objective of this research was to produce barrier membranes from biodegradable polymers, namely, PLLA and PCL, with an antibacterial feature promoted by propolis. The structure and properties of the cast films with/without propolis were investigated using various techniques.

## 2. Materials and Methods

### 2.1. Materials

Poly(L-lactide) was kindly donated from Corbion-Purac (Purasorb® PL24, The Netherlands) and used as obtained. Poly(ε-caprolactone) (MW = 80000) and chloroform were obtained from Sigma (Germany). Propolis was provided by the local beekeepers.

### 2.2. Preparation of Ethanol-Extracted Propolis

Propolis was extracted in 95% (v/v) ethyl alcohol in a hermetically closed glass container at 37°C for 4 h under shaking. The extract was then filtered with a filter paper (Whatman No. 4) and dried under vacuum until ethanol is completely removed. The dried extract was lyophilized after freezing at −20°C and kept in the dark until use.

### 2.3. Preparation of Polymer/Propolis Films

The solvent casting method was selected to prepare the composite films with different PLA/PCL/propolis ratios. To obtain the final homogenous cast solution, each component was separately dissolved in chloroform. Firstly, individual solutions of two polymers were mixed and stirred for 2 h. Propolis solution in chloroform was then added to the mixture, and the final mixture was allowed to stir for next couple of hours. The certain amount of the final polymer-propolis mixture was casted into the glass Petri dish. The Petri dishes were then dried at room temperature for 24 hours and stored at +4°C in the dark.

### 2.4. Characterization

Surface morphology of the produced films was visualized by a scanning electron microscope (SEM XL30 ESEM-FEG, FEI-Philips, The Netherlands). All the samples were sputter-coated with a layer of platinum prior to observation and then examined using an accelerated voltage of 15 kV.

To determine the chemical structure of the polymeric films and observe the structural differences between films, the FTIR spectra of films were recorded with Fourier transform infrared spectroscopy (FTIR, PerkinElmer Spectrum 100, USA).

The influence of propolis presence on the thermal behavior of the composite scaffold was investigated by DSC analysis (Seiko–SΙΙ DSC7020, Tokyo, Japan.) The samples were heated to a temperature range from −20°C to 200°C, at the heating rate of 10°C/min and cooling rate of 100°C, under the nitrogen atmosphere.

The tensile strength and elongation of the films with a size of 50 × 5 × 0.2 mm were measured with a universal test machine (Zwick/Roell, Germany). The entire testing process was conducted at a room temperature of 25°C with 70% relative humidity using 0.1 MPa forces with a constant drawing speed of 125 mm/min. Average values and standard deviations of five replicates of each specimen were reported.

The *in vitro* degradation test was carried out in phosphate-buffered saline solution (PBS) under physiological conditions (*T* = 37°C at pH = 7.4). In order to prevent contamination, 2% sodium azide (NaN_3_) was added into the solution. The solution was supplemented with *α*-amylase (140 U/L), which is the primary enzyme in saliva, to mimic the mouth inside environment. The samples (*n*=5) were soaked into PBS/amylase containing plastic tubes and incubated in an oven for 90 days. At predefined time points (15, 30, 60, and 90 days), the samples were removed from the solution, washed with distilled water, and dried at 37°C for 24 hours prior to weighing. The weight loss percentage was calculated as follows: weight loss % = [(*w*_0_ − *w*_F_)/*w*_0_] × 100%, where *w*_0_ and *w*_f_ are the initial and final dry weights of the sample, respectively.

The antibacterial activity test was performed on Mueller-Hinton agar (MHA) using the disk diffusion method by the “zone inhibition” test according to standards published by the Clinical and Laboratory Standards Institute (CLSI) for bacteria testing [22]. *Staphylococcus aureus* ATCC 29213 was used to test the antibacterial activity of sample disks. Each sample disk was placed on a part of the Petri dish which was divided into 4 parts. Additionally, a Petri dish was used for an antibiotic disk including 30 *μ*gr chloramphenicol (C30) (HIMEDIA) and without propolis as a positive control and a negative control, respectively. In brief, Gram (+) bacteria *S. aureus* was cultured in the 2 ml of Mueller-Hinton broth with a shaking rate of 170 rpm at 37°C overnight. After that, the cell density was adjusted 0.5 McFarland by the turbidity meter in sterile physiologic saline water (PBS) (0.85 w/v). 100 *μ*l cell suspension in PBS of 0.5 McFarland *S. aureus* was inoculated on MHA by a sterile swab. Sample disks were gently placed on inoculated MHA Petri dishes. Later, inoculated Petri dishes were incubated at 37°C and for 24 h. Antibacterial assay was performed in at least triplicate. After the incubation period, Petri dishes were visualized by an imaging system (Vilber-Lourmat Imaging System), and the diameters of the inhibition zone were measured as millimeters.

## 3. Results and Discussion

### 3.1. Surface Morphology of the Films

**Figure 1:**
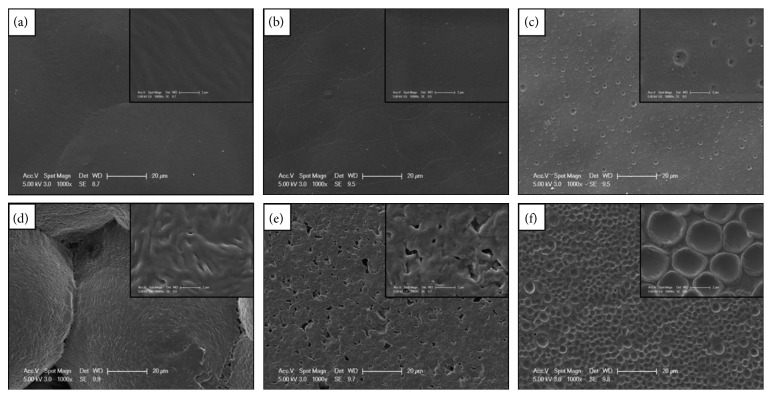
SEM micrographs of the surface of the produced film: (a) neat PCL; (b) neat PLLA; (c) PLLA9-PCL1; (d) PCL/P; (e) PLLA/P; (f) PLLA9-PCL1/P.

Surface properties of biomaterials are most important term for the evaluation of the body response against the material. The surface roughness of the material plays a crucial role in the regulation of selective cell adhesion and proliferation to the implant surfaces. For instance, sensitivity of the osteoblast cell to surface roughness has been discussed extensively in the literature [23, 24]. Considering that the films produced in this work will contact with the osteoblast and fibroblast cells *in vivo*, the surface topography of the films was examined by SEM.  Figure 1 presents the SEM images from the produced composite film with different component ratios. It was revealed that PCL and propolis divert the smoothness of the PLA film surface. The presence of PCL caused little pits on the upmost layer of the PLA film due to their solubility difference in the chosen solvent. Although PCL is extensively preferred to improve fracture energy of PLA, they form immiscible blends [25–27]. The irregularity of the surfaces was increased even more with the addition of propolis to neat the polymer or polymer blends (Figure 1). When the blends of propolis with neat polymers were evaluated, the phase separation between PCL and propolis was found to be much higher than PLLA and propolis. The surface of the three-component film, PLLA/PCL/P, presents honeycombed-like surface irregularities that may possibly change the cell response to these hydrophobic polymer films in a positive manner.

### 3.2. FTIR

**Figure 2:**
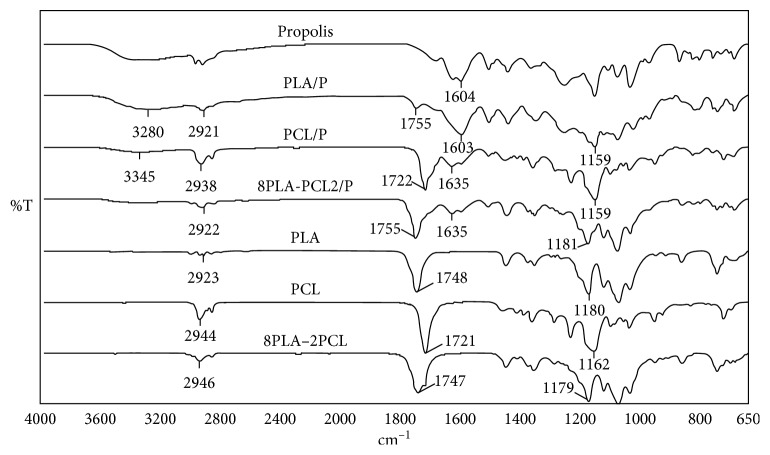
FTIR spectra for the films without/with propolis.

The structure of polymeric films was investigated using FTIR spectroscopy (Figure 2). In the spectra of the neat PLLA film, major characteristic bands C-H stretching, C=O band, and C-O band appeared at 2923 cm^−1^, 1748 cm^−1^, and 1181 cm^−1^, respectively [28]. The PCL film, which has a similar chemical structure with PLLA, C-H stretching, C=O band, and C-O band, was observed at 2944 cm^−1^, 1721 cm^−1^, and 1162 cm^−1^, respectively. In case of the three-component film, PLLA8/PCL2/P, same bands were occurred in 1179 cm^−1^ (C-O band), 1747 cm^−1^ (C=O band), and 2946 cm^−1^ (C-H stretching), while the characteristic bands of propolis were also observed at 3358 cm^−1^ (O-H vibration) and 1641 cm^−1^ (C=C double bond from the flavonoid component) [29–31]. Besides these, the intensity of the C=O group of PLLA dramatically decreases in the presence of propolis, whereas there was no similar changes in the PCL-propolis mixture, which compromise with SEM results regarding the difference on the compatibility of propolis with the polymers.

### 3.3. Thermal Properties

The DSC results of films are given in Table 1. The neat PLLA film has *T*_g_ and *T*_m_ values of 52.3°C and 180°C, respectively, whereas PCL exhibits *T*_m_ at 62.5°C. For both of polymers, these values are consistent with literature. In the blend form, the melting points of PCL increased with increasing amount of the PLLA content. On the contrary, the presence of PCL showed no influence on the *T*_m_ of PLLA due to the difference on the crystalline structure formation of PLLA and PCL as reported by the others [32]. The presence of propolis significantly affected the thermal properties of films. Both polymers showed around 15°C of a decrease in melting temperature in the films with propolis. There was a drop in *T*_g_ of about 14.4°C for PLLA. Propolis has a soft and sticky character between 25 and 45°C, becomes more stickier above 45°C, and transforms into the liquid phase at 60–70°C. Propolis is a rather small molecule compared to polymers and can be easily located between huge polymer molecules. Therefore, it limits the interactions between polymer chains and finally causes decreases in the melting temperature of polymers. This decrease was more significant in the PLLA/PCL/propolis blends. Moreover, it was not possible to observe a sharp melting peak of PCL in three-component blends due to the overlap with propolis liquid-phase transformation.

### 3.4. Mechanical Properties

Elasticity and mechanical durability are crucial parameters in the evaluation of the relevance of a barrier membrane for GTR applications. The membrane should withstand with the tissue tension to prevent collapse of soft tissue into the wound site in order to maintain the space for regeneration. Besides that, some extrinsic factors such as membrane handling characteristics and ease of placement also indirectly affect the success of the treatment. In the present study, we tried to achieve the desired mechanical properties to take the advantage of mechanical strength of PLA/PCL blend and the waxy nature of propolis. The results obtained from tensile tests are given in Table 2. It was observed that the addition of propolis causes a 3-4-fold decrease in tensile strength, whereas the film elongation values increase significantly in the presence of propolis. This may be due to the propolis interference on polymer chain orientation during the solidification process of blend solution. The similar results have been reported for polyurethane membranes by Kim et al. and gelatin-based films by Bodini et al. [33, 34]. The increase in the elasticity enhances the folding and handling ability of the membranes, which allows the clinicians to work easier in 3D effects.

### 3.5. *In Vitro* Degradation Studies

**Figure 3:**
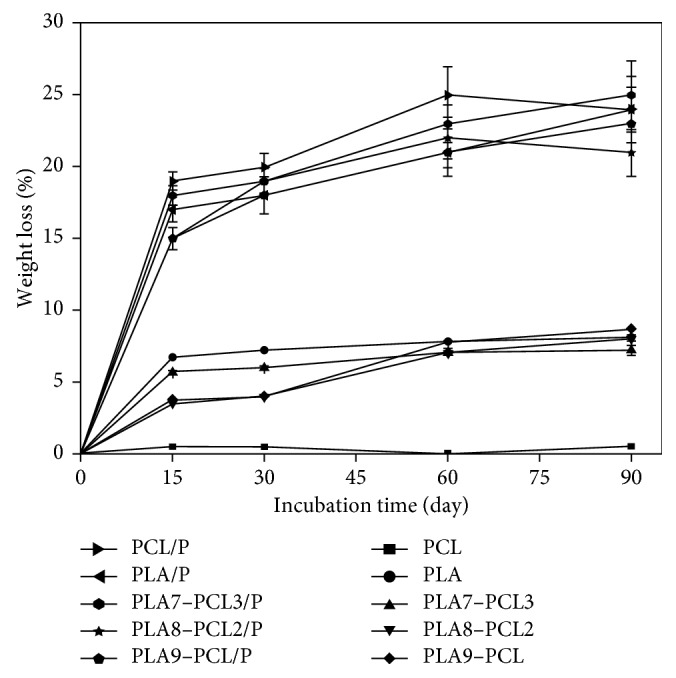
Weight loss of the produced film after incubation in degradation solutions containing *α*-amylase at 37°C (*n*=3).

Figure 3 shows the degradation behavior of prepared films at pH 7.4 in the presence of *α*-amylase in terms of weight loss. All samples exhibit a rapid weight loss during the first week, and then the sample mass tends to decrease slowly in the following weeks. Comparing the degradation profile of the propolis containing films, the lost weight was found to be between 20 and 25 wt.% at the end of the test, whereas it was around 8 wt.% for the films without propolis, except neat PCL. These results confirmed one more time the disruption in the crystalline structure of the polymers caused by propolis.

### 3.6. Antibacterial Properties

**Figure 4:**
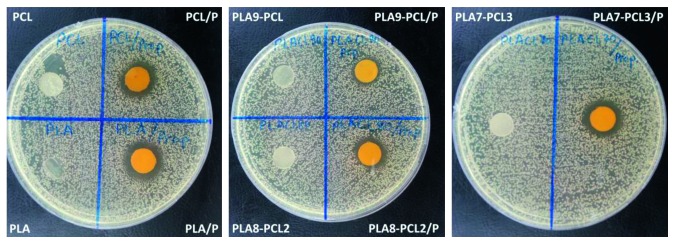
Zone of inhibition formed by films against *Staphylococcus aureus.*

The motivation of this study is to provide an antibacterial property to the biodegradable polymer films using a natural and nontoxic additive. The mechanisms of action underlying the antibacterial effects of propolis have been widely described in the literature [30, 35]. The possible mechanism is related to cinnamic acid and flavonoid components of propolis which make changes on the ion permeability of the inner bacterial membrane causing the dissipation of the membrane potential and inhibition of bacterial motility [35]. Herein, we tested the antibacterial properties of the films against *Staphylococcus aureus*, which is a common type of bacteria in the oral environment. Regarding the results presented in Figure 4, the bacterial growth was inhibited around propolis containing films, while they were proliferated around the polymer film without propolis. There was no significant difference on the widths of the inhibition zones since the amount of propolis for each film was kept constant. Therefore, the conclusion can be drawn that the addition of the accurate amount of propolis into the polymeric films is an effective method to provide antibacterial properties to the films (Table 3).

## 4. Conclusion

PLLA/PCL/propolis films were successfully fabricated using the solvent casting method for GTR application. Propolis changed surface topography of the films and created honeycomb-like structures, which could be beneficial for osteoblast cell adhesion. It has also positive influence on the thermal, mechanical, and degradation properties of the blend films to achieve the required values for GTR. Moreover, films with propolis showed antibacterial activity against Gram (+) bacteria. Our findings indicate that PLLA/PCL/propolis composite films can be used as an antibacterial biodegradable barrier membrane for guided tissue engineering application.

## Figures and Tables

**Table 1:** DSC results of films without/with propolis.

	*T*_g_ (°C)	*T*_m_ PLA (°C)	*T*_m_ PCL (°C)
PCL	−60	—	62.52
PCL/P	−23.10	—	47.08
PLLA7-PCL3	46.57	178.86	52.15
PLLA7-PCL3/P	43.87	161.85	68.07
PLLA8-PCL2	42.85	178.79	54.24
PLLA9-PCL1	44.64	178.99	57.32
PLLA9-PCL1/P	34.61	165.02	39.44
PLLA	52.32	180.03	—
PLLA/P	37.94	164.08	—

**Table 2:** Mechanical properties of films without/with propolis.

	*E*_T_ (MPA)	*ɛ*_M_ (%)
PCL	247 ± 8	980 ± 94
PCL/P	20.5 ± 7	320 ± 9
PLLA7-PCL3	339 ± 63	160 ± 14
PLLA7-PCL3/P	122 ± 6	130 ± 21
PLLA8-PCL2	684 ± 27	220 ± 25
PLLA8-PCL2/P	319 ± 13	133 ± 16
PLLA9-PCL1	818 ± 9	240 ± 19
PLLA9-PCL1/P	336 ± 11	200 ± 15
PLLA	829 ± 17	170 ± 2
PLLA/P	226 ± 10	210 ± 4

**Table 3:** Antibacterial activity of the prepared films.

	(+) control	(−) control	PLLA/P	PCL/P	PLLA7-PCL3/P	PLLA8-PCL2/P	PLLA9-PCL1/P
Antibacterial activity	+	−	+	+	+	+	+
Inhibition zone diameter (mm)	27	−	17	15	17	13	13

## Data Availability

The data used to support the findings of this study are available from the corresponding author upon request.
